# Fitness integrated with technology approach to teaching biomechanics and STEM in a high school setting: a case report

**DOI:** 10.3389/fspor.2025.1681868

**Published:** 2025-10-21

**Authors:** Stuart Evans, Charlene Willis

**Affiliations:** ^1^ School of Education, La Trobe University, Melbourne, VIC, Australia; ^2^ School of Pharmacy and Medical Sciences, Griffith University, Brisbane, QLD, Australia

**Keywords:** STEM, STEM - science technology engineering mathematics, physical education (PE), high school, biomechanics

## Abstract

Many high school students withdraw from physical education (PE) and sport science majors (including biomechanics) due to anxiety about science and mathematics. In some situations, it is not necessarily what STEM subject is taught but how it is introduced and what pedagogy is applied by the teacher. In high school, students who study PE are often required to understand basic biomechanical principles. Initial research supports the positive effects that action-based and game-based pedagogy has on students’ attitudes toward the field of biomechanics; however, quantitative evidence remains sparse. This is particularly true when wider STEM integration is considered. The purpose of this study was to quantify high school students’ (*n* = 14) perceptions toward biomechanics after participating in a Fitness Integrated with Technology (F.I.T) and a tactical games approach (TGA) over 16 lessons (eight weeks in total). Professional development workshops were used to help the teacher develop pedagogical content knowledge to teach biomechanics using the F.I.T and TGA approach. As a primary outcome measure, the Student Assessment of Learning Gains (SALG) instrument was used pre and post the F.I.T. theoretical and practical lessons while variables including the feasibility, practicality, and challenges of applying F.I.T. was used as a secondary measure. This was applied to better understand the impact of using F.I.T and TGA to teach an integrated STEM and biomechanics subject. SALG scores exhibited a significant difference pre and post the F.I.T approach (*p* < 0.001) with the biggest changes seen in the “excellent learning gain” category post the F.I.T. implementation (*t* = −2.26, *p* = 0.0019) demonstrating that students felt they had made satisfactory to very good learning gains by the end of the final lesson. These findings further support the ability for F.I.T and TGA approaches to positively impact students’ perceptions toward biomechanics and STEM, although opportunities persist to increase student career interest in both STEM, PE and biomechanics. This case report presents and discusses the study’s results, interpretations, limitations, and implications for future research on integrated biomechanics and STEM outreach activities.

## Introduction

Many high school students withdraw from physical education (PE) and sport science majors (including biomechanics) due to anxiety about science and mathematics. In some situations, it is not necessarily what STEM subject is taught but how it is introduced and what pedagogy is applied by the teacher. In high school, students who study PE are often required to understand basic biomechanical principles. Initial research supports the positive effects that action-based and game-based pedagogy has on students’ attitudes toward the field of biomechanics; however, quantitative evidence remains sparse. This is particularly true when wider STEM integration is considered. The purpose of this study was to quantify high school students' (*n* = 14) perceptions toward biomechanics after participating in a Fitness Integrated with Technology (F.I.T) and a tactical games approach (TGA) over 16 lessons (eight weeks in total). Professional development workshops were used to help the teacher develop pedagogical content knowledge to teach biomechanics using the F.I.T and TGA approach. As a primary outcome measure, the Student Assessment of Learning Gains (SALG) instrument was used pre and post the F.I.T. theoretical and practical lessons while variables including the feasibility, practicality, and challenges of applying F.I.T. was used as a secondary measure. This was applied to better understand the impact of using F.I.T and TGA to teach an integrated STEM and biomechanics subject. SALG scores exhibited a significant difference pre and post the F.I.T approach (*p* < 0.001) with the biggest changes seen in the “excellent learning gain” category post the F.I.T. implementation (*t* = −2.26, *p* = 0.0019) demonstrating that students felt they had made satisfactory to very good learning gains by the end of the final lesson. These findings further support the ability for F.I.T and TGA approaches to positively impact students' perceptions toward biomechanics and STEM, although opportunities persist to increase student career interest in both STEM, PE and biomechanics. This case report presents and discusses the study's results, interpretations, limitations, and implications for future research on integrated biomechanics and STEM outreach activities.

## Background

Many high school students may worry and dislike basic biomechanics, which can add to the challenges teachers face in teaching and supporting student learning. There is evidence that dynamic and participative learning methodologies can improve student engagement with knowledge of biomechanical concepts. To improve academic performance, large improvements in learning have been reported for active learning approaches compared to the traditional lecture (1). Moreover, the evidence for greater student engagement due to active learning across many fields of education and science is compelling (2).

The Internet of Things (IoT) has pushed wearable technology integration into many educational disciplines. Nevertheless, the acceptance in high school PE has been slower. Physical activity has been found to produce improvements in children's executive function, including attention and self-control (3), which is an important foundational skill for STEM (4). From game-day analytics to injury prevention and equipment design, students can instinctively and naturally connect STEM concepts to real-world sports, fitness, and health scenarios. However, despite the emphasis on teaching STEM, few PE teachers feel prepared to integrate approaches for STEM instruction (5).

Biomechanics is the application of mechanical principles to the study of living organisms. Specifically, it combines engineering, physics, and mathematics together with biology to understand movement (6). Yet biomechanics tends to suffer from a lack of exposure (7) and is not an apparent field of study or career path. Thus, this case report documents a pre-post methodology that examines a high school teacher's perceived assessment of students' understanding of the biomechanics of a free throw in basketball.

STEM education is an approach that presents subjects in an integrated and interdisciplinary way. The definition of STEM varies significantly according to the educational level (8) and the student cohort. At the secondary education level (i.e., high school) science and mathematics are commonly integrated into specific science fields such as physics, advanced mathematics and chemistry. The importance of STEM at these levels lies in the fact high school education plays a fundamental role in students' decision to consider a STEM-related career education (9) as the need for qualified STEM graduates has repeatedly been stressed by different governmental and academic institutions globally (10).

The importance of STEM graduates is commonly accompanied with a tagline analogous to “today's students are needed to fill the jobs of tomorrow”, despite the relative unknown of what tomorrow's jobs may be. Recent data show a global decline in STEM careers (11), yet Honey et al. (12) demonstrated that studying these subjects in an incorporated way increases learning outcomes and student motivation to learn. Similarly, Sanders (13) found that interdisciplinary STEM education fosters a deeper understanding of the interconnectedness of scientific and technological advancements, preparing students to address global challenges. Therefore, providing pedagogically robust learning experiences in biomechanics to high school students is important not only for the future generation of physical educators and biomechanics researchers but also for doctors, engineers, rehabilitation personnel, movement scientists, ergonomists, any health-related professions.

Attempts have been made to improve PE classes in response to rapidly changing societies. Since Carlson's study in 1995, there has been growing interest in the problem of students who avoid PE and are alienated from it (14). Alienation in PE arises in two ways. The first involves students just sitting in class meaninglessly because they are not interested in PE itself. The second involves students participating in the class at the beginning and then losing interest midway through, whereafter they become indifferent to class activities (15). Today's knowledge-based society is that of a person who does not merely hold exponentially increased knowledge and information but can creatively solve complex problems they face by themselves or in a cooperative relationship (16). Thus, there is a need for teaching and learning that can increase the core competency of self-directed learning.

The Victorian Certificate of Education (VCE) is the senior secondary qualification awarded to high school students in Victoria, Australia, upon successful completion of high school secondary education. It is recognized both locally and internationally, and students typically complete it over the final two years of high school (17). VCE PE allows students the opportunity to participate in a range of physical activity opportunities aimed at applying theoretical concepts that promote understanding of areas such as anatomy and physiology, biomechanics, and performance enhancement. Notably, VCE PE encourages teachers to adopt an interdisciplinary and integrative approach to teaching. Yet like students, teachers can learn through informal interactions that occur during peer teaching, collaborative planning, and mentoring between colleagues (18).

## Case history

A case of a male teacher (age: 34; teaching experience: 8 years) based at an inner-city culturally and linguistically (CALD) diverse school in Melbourne, Australia, who had 14 high school students (8 male, 6 females; age 15 ± 0.49) all in their first year (grade year 11) of studying high school VCE PE, is described. In July 2024 (Term 3), the teacher was scheduled to teach VCE PE, focusing on biomechanics. The teacher and students volunteered to be part of this case study. Exclusion criteria included students not being taught by the male teacher or those not studying VCE PE. The teacher was required to teach multifaceted interrelationships between biophysical and psychosocial concepts to enable students to understand their role in producing and refining movement for participation and performance in physical activity, sport and exercise. His teaching commenced in July 2024 for 10 weeks, although biomechanics and the biophysical and psychosocial concepts associated to human movement were taught for eight weeks. The teacher had minimal experience in teaching VCE PE and had no prior experience in teaching biomechanics nor using an interdisciplinary approach. Similarly, his students had no prior knowledge of calculus or primitive biomechanical formulas and therefore the teacher used example videos of sporting performance as well as practical demonstrations to highlight some of the challenging components that needed to be taught (i.e., angular acceleration).

In May 2024 he had undertaken self-directed learning by way of a two-hour professional development workshop that was facilitated by an external education provider. He requested assistance in developing his pedagogical content knowledge in biomechanics and how to apply STEM components into his teaching due to the necessity for students to learn mathematical and scientific principles while engaging and utilizing technology.

From May 2024 to July 2024 the teacher attended five online professional development workshops, each lasting for approximately 60 min that were facilitated by the researchers, in biomechanical principles, game-based pedagogy and interdisciplinary approaches to teaching. The workshops were designed to provide the teacher with background knowledge in subject specific content and curriculum coherence that would allow integrative theoretical and practical lessons that met learning intentions. The teacher took notes throughout the workshops. In July 2024 the teacher presented a scope and sequence document (Supplementary Appendix A) to the researchers that outlined how lessons would be integrated and taught that aligned with the VCE PE curriculum and that were pedagogically adaptive to the subject topic.

In July 2025 his students returned to school after a two-week mid-semester break. The teacher's first lesson with the students was a classroom-based lesson that occurred on the first Tuesday of Term 3, week 1. Lessons were held either in a classroom or in a conventional indoor and outdoor basketball court. A total of two lessons a week were delivered for eight weeks (i.e., 16 lessons in total). Both theoretical and practical lessons lasted for 50 min. The classroom (theoretical) lesson was held each Monday from 0900 to 0950 with the practical lessons timetabled for Wednesday at 1,100–1,150. The purpose of the theoretical lessons was to allow the teacher to deliver appropriate weekly content that explained the biomechanical principles related to the countermovement jump and standing vertical jump, sport specific movements selected by the teacher. This multifaceted approach allowed students to make tangible connections between theory and practice.

The researchers assisted the teacher to structure the classroom content to contain the biomechanical variables including stationary standing, unweighting, propulsion, and take off. The curriculum components comprised power, Newtonian physics, force (F); vertical velocity (v); acceleration (a); mass (m); and displacement of COM (_d_com). For example, the concept of mass was defined as the resistance (force) opposed by a body to being accelerated. The COM was defined as the point in which the mass of a body could be concentrated to simplify the analysis of movement or as its projection on the floor. The learning objectives aligned to VCE requirements, with a focus on Newton's Laws. Specifically, lessons were designed to help students understand the fundamental principles of motion and forces inclusive of relating balanced forces to motion states, connecting acceleration to net force, defining mass and weight and identification of forces acting upon an object (Table 1).

**Table 1:** Lesson principles and instructional approaches.

Lesson and principle	Unit 1: The Human body in Motion (Victorian Certificate in Education, VCE PE) Unit 3: Movement skills and energy for physical activity, sport and exercise (Victorian Certificate in Education, PE)	Fitness Integrated with Technology (F.I.T). and STEM component
Week 1–2: stationary standing	Topic: STEM in sports (STEM in basketball)	• Technological integration and engineering dynamics of Human Activity Monitor (HAM). • Science of movement and measurement pertaining to jumps • Newton's First Law of Motion • Introduction to weight (force expressed in Newton (N) relationship to body mass (expressed in k.gm) multiplied by *g* (acceleration of gravity expressed in m/s^2^.)
Week 3–4 unweighting	Topic: application of STEM in basketball	• Technological integration and engineering relevance • Science of movement and measurement, exploration of ground reaction force (GRF) and links to Newton's Third Law • Newton's Second Law of Motion
Week 5–6 propulsion	Topic: STEM applications in internal (COM) and external (GRF) contexts.	• Technological integration and engineering relevance • Science of movement and measurement. Principles of COM acceleration. • Newton's Second and Third Laws of Motion
Week 7–8 take off and combined motion.	Topic: STEM consolidation	• Consolidation of learning and practical combination of Newton's Three Laws of Motion. • Overall links to STEM and F.I.T. assessed by SALG instrument.

**Figure 1:**
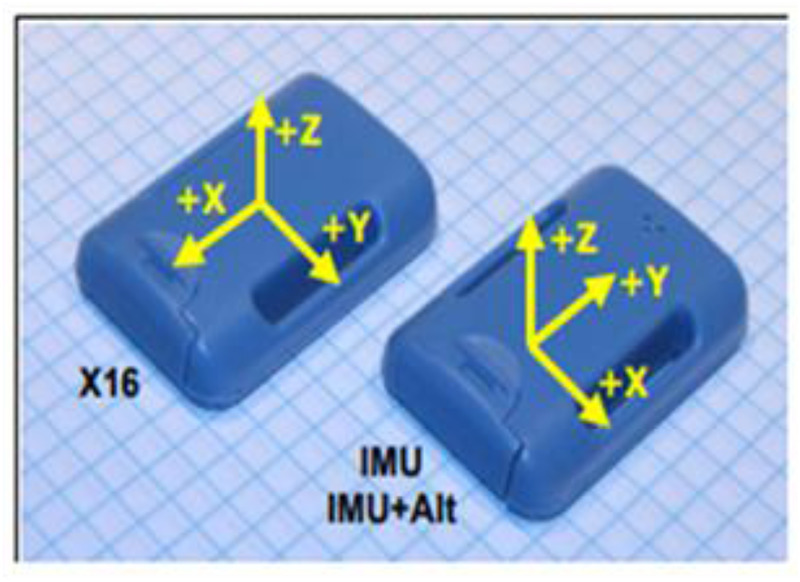
HAM with axis of orientation (leftmost) (*x*, upward–downward), anteroposterior (*z*, forward–backward), and mediolateral (*y*, side to side). Where X16 is the model number; IMU is inertial measurement unit; Alt is alternate navigation.

The teacher used a Tactical Game Approach (TGA) in all practical lessons, incorporating the theoretical classroom components listed in Table 1. The TGA approach was constructed around the TGA framework (19) which comprised four key components: (a) an initial modified game; (b) tactical questioning to enhance understanding and application; (c) skill explanations followed by practice; and (d) a concluding modified game to reinforce learning. In each practical lesson the teacher organized the students into two groups in which each student wore a human activity monitor (HAM). The HAM (Gulf Coast Data Concepts, Waveland, MS) (Figure 1) is a compact self-recording data logger with 6 degrees of freedom. The HAM (5.5 × 4.5 × 3.5 cm, 10 g) was manually initialized to record raw accelerations at a sampling frequency of 50 Hz. The HAM was placed on the student's lumbar five (L5) sacrum one (S1) position. Alignment of the sensor captured data in three orthogonal directions, specifically in the vertical (x, upward–downward), anteroposterior (z, forward–backward), and mediolateral (y, side to side) (in gravitational acceleration, g, where 1 g is equal to 9.81 m/s^2^). The HAM was calibrated as per manufacturer's instructions, that is - under nonmovement conditions using local gravitational acceleration as a reference.

**Figure 2:**
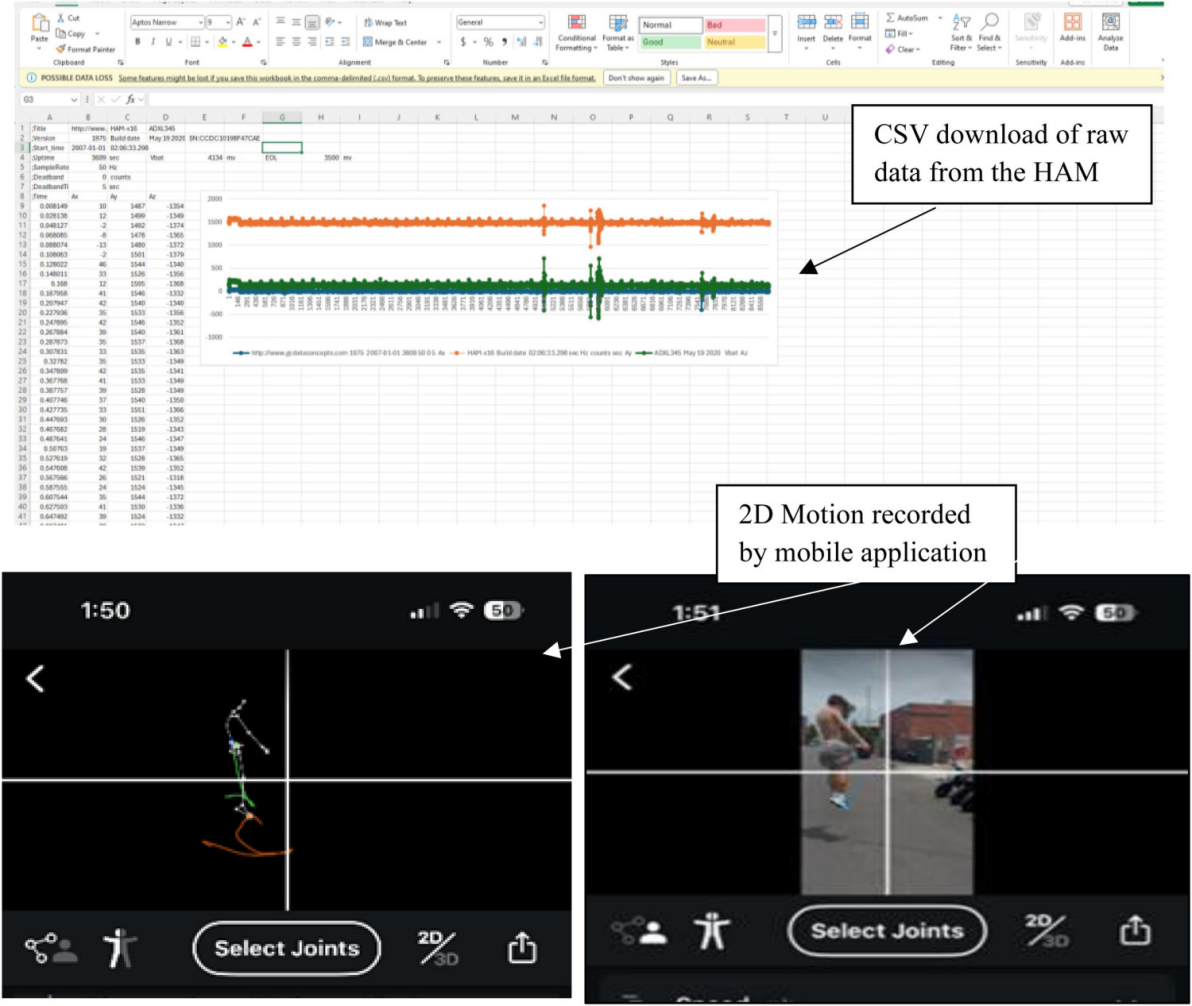
Topmost: raw data obtained via the HAM. Bottommost: 2D SPLYZA motion data.

In groups, and when asked by the teacher, students collaborated by using a markerless mobile application (SPLYZA Motion, Hamamatsu City, Shizuoka, Japan) to record each other's countermovement and vertical jump during the TGA - that is, one student performed the countermovement jump and vertical jump while wearing a HAM while another recorded the motion via their own smart phone device. Raw acceleration data from the SPLYZA Motion MMC was recorded at a sampling rate of 60 Hz. At the end of the practical lessons, raw data from both systems were downloaded and processed by the students in CSV format and saved and exported to Microsoft Excel (Microsoft Corporation, Redmond, Washington, USA, Version 16). The data was then deconstructed into three second epochs by the students to consolidate the timestamp between the two systems. This data was used by the students in the theoretical classes whereby they analysed, interpreted and applied graphical, visual and physical representations of the jumps by looking at both HAM and SPLYZA data sets (Figure 2). Data from the HAM and SPLYZA were then superimposed on a single graphical representation to produce an overlay.

The teacher applied scaffolding techniques where appropriate to assist with student interpretation and comprehension of the data. The teacher then used a Student Assessment of Learning Gains (SALG) instrument as a method of analysis pre and post the theoretical and practical F.I.T. lessons. The SALG consists of statements about the degree of “gain” (on a five-point scale) in specific aspects of the class. Specifically, the SALG seeks to aggregate data on student-reported learning outcomes (20) within content areas including student understanding, skills, cognition, attitudes, integration of learning, and motivation toward the learning in areas identified as relevant to the learning activities. Within each category of SALG questions, students provided quantitative ratings on statements about the degree to which specific lesson attributes supported or contributed to their learning and the approaches taken to teach the subject. These items were scored on a Likert scale ranging from 0 to 4 where 0 = no help/gain to 4 = excellent help/gain. The total SALG score and each subscale score was then calculated and divided by the number of items therein. The SALG instrument was completed anonymously by the students.

Statistical analyses were completed by SPSS version 20 (SPSS Inc., Chicago, IL, USA). Descriptive statistics included frequencies, measures of central tendencies and variability. Due to the non-normal distribution of data, a Mann–Whitney U and Cronbach's alpha reliability (21) was used. A Student's *t*-test was used to assess pre and post the F.I.T along with Cohen's *d* effect size with conventions small (*δ* = 0.2), medium (*δ* = 0.5), and large (*δ* = 0.8) (22). The level of significance was set at 0.05.

Student attendance throughout the 8-weeks was 94% with overall fidelity of the F.I.T and TGA pedagogies retained. A Cronbach's alpha reliability of 0.7 was recorded which indicated reliability. Upon cessation of the lessons, SALG scores were aggregated which exhibited a significant difference pre and post the F.I.T approach (*p* < 0.001) with the biggest changes seen in the “excellent learning gain” category post the F.I.T. implementation (*t* = −2.26, *p* = 0.0019) demonstrating that students felt they had made satisfactory to very good gains in their learning by the end of the final lesson (Table 2). Other notable changes to learning included a shift from satisfactory gains (pre F.I.T.) to very good gains (post F.I.T).

**Table 2:** Anonymous student responses (*n* = 14) of SALG scores of pre-post F.I.T. intervention. Where *t* is the student's *t*-test and *d* is Cohen's effect size.

Status	A little gain	*t*	*d*	Satisfactory gain	*t*	*d*	Very good gain	*t*	*d*	Excellent gain	*t*	*d*
Pre F.I.T.	8 (±0.7)	3.27	0.79 (medium)	8.1 (±0.2)	2.53	0.71 (medium)	3.2 (±0.4)	1.0	0.62 (medium)	1 (±0.1)	2.2	0.81 (medium)
Post F.I.T.	0.1 (±0.5)			2 (±0.1)			5.1 (±0.5)			10.5 (±0.4)		

**Figure 3:**
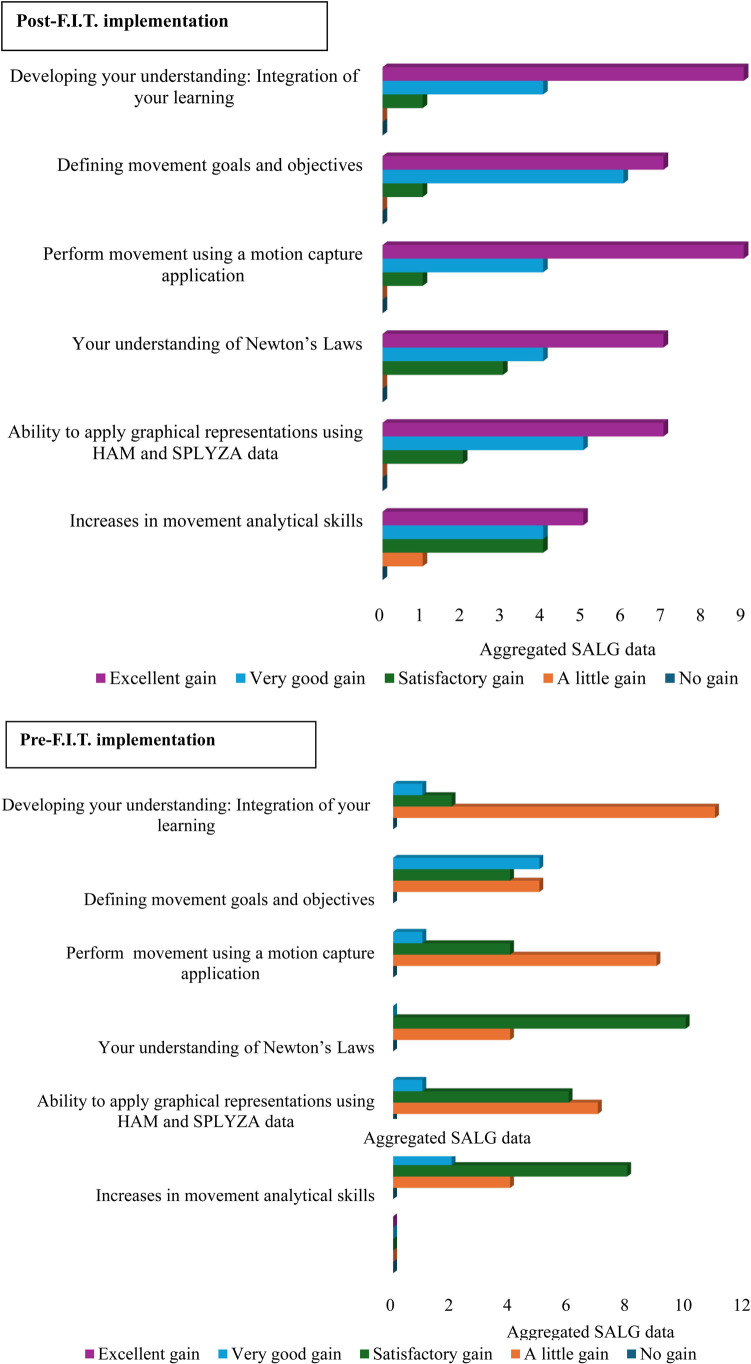
Topmost: SALG scores post F.I.T implementation. Bottommost: SALG score pre–F.I.T implementation. Mean SALG score for each subsection of the SALG questionnaire (*n* = 14). Score 0 = help/gain; 1 = a little help/gain; 2 = satisfactory help/gain; 3 = very good help/gain and 4 = excellent help/gain.

While all items of the teaching and learning approaches were positively evaluated, the greatest enablers were *performing movement using a motion capture* system (*p* < 0.01) followed by the *developing understanding and integration of learning* (*p* < 0.01) (Figure 3).

## Discussion

Instruction and learning in STEM remain an educational priority for many countries. In recent years, the need to expand STEM education has been framed in the context of preparing students for future jobs (23) and ensuring students develop problem-solving skills to address current and future problems in the world (24). Advocates of STEM contend that critical STEM thinking is essential. While some researchers have explored how STEM can be integrated into social studies (25), music (26), and arts (27), its integration into biomechanics and high school PE remains relatively unresearched.

The teacher in this case study (to the authors' knowledge) had no prior experience of STEM integration within a PE and sport science subject discipline. While research on STEM in schools indicates that it is possible to integrate STEM curricula across disciplines (28) high school teachers’ perspectives are seldom reported. Moreover, to the authors' knowledge this is the first use of the SALG metric to measure learning gains in interdisciplinary high school biomechanics and STEM educational research from a teacher and student perspective. Accordingly, the SALG instrument is a relatively easy-to-use tool that teachers can use to perceive learning gains identified as important.

Despite initial apprehension, the teacher and students appreciated the incorporation of wearable technology, yet the teacher acknowledged that barriers existed in implementing technology in PE and teaching to wider STEM principles. Our teacher is like others in that a teacher's ideology can influence the role that technology will play in their teaching (29), and as Casey et al. (30) argue, it is important that opportunities for digital technologies are explored to positively shape PE and STEM. Moreover, use of surface electromyography (sEMG) to measure muscle activity, largely associated to biomechanics, may elicit curiosity and fun and extend students' knowledge in how additional STEM principles can be applied.

This case report does present limitations, including the small sample size and the use of self-reporting measures that relies on observer effects. It should be noted that the teacher assisted the students by way of scaffolding learning to maintain curriculum alignment. Yet scaffolding is pedagogically appropriate and valid, particularly technology-enabled feedback, the latter perceived as an excellent gain. Support of learning gains may also be due to several factors as outlined by Haraldseid et al. (31), inclusive of physical, psychosocial, and possibly organizational factors. However, this provides further scope for research, notably by using a control group with a larger cohort that includes a mixture of primary, middle and high school students. Despite this, based on the observations made in this novel case report, there is strong justification for high school teachers to use a F.I.T and TGA based approach that utilizes wider STEM principles to support students to understand fundamental biomechanical principles. While this approach represents a shift from traditional teaching approaches, the use of interdisciplinary approaches could be extended to different year groups and subjects. Such an approach could also be extended to higher education where sport and exercise science degree programs may benefit from the use of technology as a way to deliver practice-oriented activities through distance teaching without compromising learning objectives (32).

## Teacher perspective

“I was not convinced about how this approach would work. I do not have much time with the students per week, so any wasted time is precious. Importantly, for students at this age they need to see the benefits otherwise they will become distracted and lose interest. It was a bumpy process at first as I needed to understand how the component parts worked. This meant having understanding and confidence that the technology worked and getting the equipment onto the students without them or me spending too long mucking around with it.

I needed to see the “big picture” and how the STEM approach would work. The learning intention had to be explicit yet fun, rewarding and engaging. Interdisciplinary, or integration of subjects, was new to me and is not something I was overly familiar with. It was a learning curve. For the students, they had to see and understand how STEM is part of what we do in PE and biomechanics. During the lessons the students were able to make the explicit links between theory and practice, often providing ideas and demonstrating their understanding of some of the more complex terms. Getting STEM to work in an integrated, or multidisciplinary way, was challenging at first, but became easier against the backdrop of sport and sporting analysis. They (the students) understood this and was reflected in their learning. I will be very happy to work using this approach again, particularly if assessments were incorporated into the learning design.”

## Data Availability

The raw data supporting the conclusions of this article will be made available by the authors, without undue reservation.

## Supplementary material

Supplementary Appendix A Scope and sequence VCE mapping document.
