# Supplementary material for: Fitness integrated with technology approach to teaching biomechanics and STEM in a high school setting: a case report
Source: Front Sports Act Living. 2025 Oct 21;7:1681868. doi: 10.3389/fspor.2025.1681868 (PMC12583150; doi:10.3389/fspor.2025.1681868)
Supplement: Supplementary Appendix A — Scope and sequence VCE mapping document. [file Table1.docx]

| Level 5 and 6 | Levels 7 and 8 | | Levels 9 and 10 (prior knowledge) |  |  |
| --- | --- | --- | --- | --- | --- |
| **Movement and Physical Activity** |  | |  | **Mapped to VCE** |  |
| **Moving the body** |  | |  |  |  |
| Practise specialised movement skills and apply them in different movement situations in indoor, outdoor and aquatic settings | Use feedback to improve body control and coordination when performing specialised movement skills | | Perform and refine specialised movement skills in challenging movement situations | **Unit 4 Area of Study 3: Using integration to inform an interdisciplinary approach**  Unit 4 Area of Study 3 provides an explicit opportunity for students to integrate theory and practice that enables them to analyse the interrelationships across Units 3 and 4 between skill acquisition, biomechanics, energy production and training concepts and the impacts these have on performance. |  |
| Design and perform a variety of movement sequences | Compose and perform movement sequences for specific purposes in a variety of contexts | | Evaluate own and others’ movement compositions, and provide and apply feedback in order to enhance performance situations |  |  |
| Propose and apply movement concepts and strategies | Practise, apply and transfer movement concepts and strategies | | Develop, implement and evaluate movement concepts and strategies for successful outcomes |  |  |
| **Understanding movement** |  | |  |  |  |
| Participate in physical activities designed to enhance fitness, and discuss the impact of regular participation on health and wellbeing | Participate in physical activities that develop health-related and skill-related fitness components, and create and monitor personal fitness plans | | Design, implement and evaluate personalised plans for improving or maintaining their own and others’ physical activity and fitness levels |  |  |
| Manipulate and modify the elements of effort, space, time, objects and people to perform movement sequences | Demonstrate and explain how the elements of effort, space, time, objects and people can enhance performance | | Analyse the impact of effort, space, time, objects and people when composing and performing movement sequences | Students reflect on their participation in either:   - a practical activity focusing on a particular movement skill, the performance of which can be compared to another individual completing the same skill - a practical activity focusing on comparing their participation in 2 different movement skills. |  |
|  | Participate in and investigate the cultural and historical significance of a range of physical activities | | Examine the role physical activity, outdoor recreation and sport play in the lives of Australians and investigate how this has changed over time |  |  |
| **Learning through movement** |  | |  |  |  |
| Participate positively in groups and teams by encouraging others and negotiating roles and responsibilities | Practise and apply personal and social skills when undertaking a range of roles in physical activities | | Devise, implement and refine strategies demonstrating leadership and collaboration skills when working in groups or teams |  |  |
| Apply critical and creative thinking processes in order to generate and assess solutions to movement challenges | Evaluate and justify reasons for decisions and choices of action when solving movement challenges | | Transfer understanding from previous movement experiences to create solutions to movement challenges | - reflect on primary data from participation in a practical activity - use primary data to demonstrate integration of theory and practice - analyse interrelationships between skill acquisition, biomechanics, energy production and training knowledge for the practical activity completed in consideration of/in relation to the impacts these have on performance between movement skills or performers. |  |
| Demonstrate ethical behaviour and fair play that aligns with the rules when participating in a range of physical activities | Modify rules and scoring systems to allow for fair play, safety and inclusive participation | | Reflect on how fair play and ethical behaviour can influence the outcomes of movement activities |  |  |
|  | | **Achievement Standard** | | | |
| By the end of Level 6, students investigate developmental changes and transitions. They understand the influences people and places have on personal identities. They recognise the influence of emotions on behaviours and discuss factors that influence how people interact. They describe their own and others’ contributions to health, physical activity, safety and wellbeing. They describe the key features of health-related fitness and the significance of physical activity participation to health and wellbeing. They examine how community wellbeing is supported by celebrating diversity and connecting to the natural and built environment.  Students demonstrate skills to work collaboratively and play fairly. They access and interpret health information. They explain and apply strategies to enhance their own and others’ health, safety and wellbeing at home, at school and in the community. They perform specialised movement skills and propose and combine movement concepts and strategies to achieve movement outcomes and solve movement challenges. They apply the elements of movement when composing and creating movement sequences. | By the end of Level 8, students investigate strategies and resources to manage changes and transitions and their impact on identities. Students evaluate the benefits of relationships on wellbeing and respecting diversity. They analyse factors that influence emotional responses. They gather and analyse health information. They investigate strategies that enhance their own and others’ health, safety and wellbeing. They investigate and apply movement concepts and strategies to achieve movement and fitness outcomes. They examine the cultural and historical significance of physical activities and examine how connecting to the environment can enhance health and wellbeing.  Students explain personal and social skills required to establish and maintain respectful relationships and promote fair play and inclusivity. They justify actions that promote their own and others’ health, safety and wellbeing at home, at school and in the community. Students demonstrate control and accuracy when performing specialised movement skills. They apply and refine movement concepts and strategies to suit different movement situations. They apply the elements of movement to compose and perform movement sequences. | | By the end of Level 10, students critically analyse contextual factors that influence their identities, relationships, decisions and behaviours. They analyse the impact of attitudes and beliefs about diversity on community connection and wellbeing. They evaluate the outcomes of emotional responses to different situations. Students access, synthesise and apply health information from credible sources to propose and justify responses to situations in the home, in the school and the community. Students propose and evaluate interventions to improve fitness and physical activity levels in their communities. They examine the role physical activity has played historically in defining cultures and cultural identities.  Students identify and analyse factors that contribute to respectful relationships. They explain the importance of cooperation, leadership and fair play across a range of health and movement contexts. They compare and contrast a range of actions that could be undertaken to enhance their own and others’ health, safety and wellbeing. They apply and transfer movement concepts and strategies to new and challenging movement situations. They apply criteria to make judgments about and refine their own and others’ specialised movement skills and movement performances. They work collaboratively to design and apply solutions to movement challenges. |  |  |
